# Hold Your Methods! How Multineuronal Firing Ensembles Can Be Studied Using Classical Spike-Train Analysis Techniques

**DOI:** 10.3389/fnsys.2019.00021

**Published:** 2019-05-17

**Authors:** Ovidiu F. Jurjuţ, Medorian Gheorghiu, Wolf Singer, Danko Nikolić, Raul C. Mureşan

**Affiliations:** ^1^Transylvanian Institute of Neuroscience, Cluj-Napoca, Romania; ^2^Technical University of Cluj-Napoca, Cluj-Napoca, Romania; ^3^Ernst Strüngmann Institute for Neuroscience in Cooperation with Max Planck Society, Frankfurt am Main, Germany; ^4^Frankfurt Institute for Advanced Studies, Frankfurt am Main, Germany; ^5^Max Planck Institute for Brain Research, Frankfurt am Main, Germany; ^6^Savedroid AG, Frankfurt am Main, Germany; ^7^Department of Psychology, University of Zagreb, Zagreb, Croatia

**Keywords:** multineuronal activity, classical spike-train analysis, tuning curve, peri-stimulus time histogram, autocorrelation, cross-correlation, visual cortex, ensembles

## Abstract

Responses of neuronal populations play an important role in the encoding of stimulus related information. However, the inherent multidimensionality required to describe population activity has imposed significant challenges and has limited the applicability of classical spike train analysis techniques. Here, we show that these limitations can be overcome. We first quantify the collective activity of neurons as multidimensional vectors (patterns). Then we characterize the behavior of these patterns by applying classical spike train analysis techniques: peri-stimulus time histograms, tuning curves and auto- and cross-correlation histograms. We find that patterns can exhibit a broad spectrum of properties, some resembling and others substantially differing from those of their component neurons. We show that in some cases pattern behavior cannot be intuitively inferred from the activity of component neurons. Importantly, silent neurons play a critical role in shaping pattern expression. By correlating pattern timing with local-field potentials, we show that the method can reveal fine temporal coordination of cortical circuits at the mesoscale. Because of its simplicity and reliance on well understood classical analysis methods the proposed approach is valuable for the study of neuronal population dynamics.

## Introduction

The microcircuitry of the neocortex is characterized by a large number of neurons each connected to thousands of afferents (Thomson and Bannister, 2003; Douglas and Martin, 2004). In this network, activity is highly distributed: the collective firing events of neurons determine the responses of their post-synaptic targets (Singer, 1999; Bruno and Sakmann, 2006). To characterize the activity of neurons collectively, concepts such as ensemble and population coding have been introduced (Schneidman et al., 2003; Johnson, 2004; Averbeck et al., 2006; Osborne et al., 2008). These concepts have received increasing attention because the extensive recurrence, complicated input-output and feedback loops in cortical microcircuits suggest complex non-linear dynamics as their *modus operandi* (Freeman, 2003; Korn and Faure, 2003; Buzsáki, 2006). Such complexity implies that many macroscopic phenomena cannot be predicted from the behavior of individual neurons but may only be observed by studying collective behavior, as demonstrated by several recent studies (Tremblay et al., 2015; Jadhav et al., 2016; Rajan et al., 2016).

Experimental techniques have provided the means for studying collective behavior through the advent of multi-electrode recordings (Gray and Singer, 1989; Erickson, 2001; Wise et al., 2004; Biederlack et al., 2006; Buzsáki, 2006) and more recently by potent imaging techniques (Harvey et al., 2012; Forli et al., 2018). These were complemented by development of multi-variate analysis methods, enabling numerous novel findings, such as: stimulus encoding in the olfactory system through cell ensemble dynamics (Friedrich and Laurent, 2001; Galán et al., 2004; Brown et al., 2005; Bathellier et al., 2008), chaotic- and attractor-like behavior of neuronal populations (Freeman, 1994), or flips among quasi-stationary states in the activity recorded from frontal areas of behaving monkeys (Abeles et al., 1995). However, many multi-variate analysis methods are not easy to implement and some of them are not necessarily well understood analytically. In addition their results may not be trivial to interpret. Classical spike train analyses on the other hand, although well understood and well known, are univariate or at best bivariate techniques, i.e., were developed for analyzing single spike trains or pairs of spike trains, respectively. Here, we propose that classical spike train analyses can also be used to characterize collective neuronal behavior. Such classical methods are easy to apply and have the advantage of being well understood and easily interpretable. In addition, they provide direct means for comparing the collective behavior of neurons to their individual behavior.

## Methods

### Experimental Procedures and Recordings

Recordings were performed in area 17 of 2 adult cats. Anesthesia was induced with ketamine (Ketanest, Parke-Davis, 10 mg kg^-1^, intramuscular) and xylazine (Rompun, Bayer, 2 mg kg^-1^, intramuscular) and maintained with a mixture of 70% N_2_O and 30% O_2_ and halothane (0.4–0.6%). Animals were paralyzed with pancuronium bromide (Pancuronium, Organon, 0.15 mg kg^-1^ h^-1^) to prevent eye movements. Glucose and electrolytes were supplemented intravenously and through a gastric catheter. The end-tidal CO_2_ and rectal temperature were kept in the range of 3–4% and 37–38°C, respectively.

Stimuli were presented binocularly on a 21 inch computer screen (HITACHI CM813ET) with 100 Hz refresh rate. To obtain binocular fusion, receptive fields were mapped for each eye and the two optical axes were aligned on the computer screen using an adjustable prism placed in front of one eye. The software used for visual stimulation was ActiveSTIM. Data were recorded with multiple silicon-based multi-electrode probes (16 channels per electrode) supplied by the Center for Neural Communication Technology at the University of Michigan (Michigan probes). A single probe consisted of four shanks (3 mm long, inter-shank distance 200 μm) each having four electrode contacts (1250 μm^2^ area, 0.3–0.5 MΩ impedance at 1000 Hz, inter-contact distance 200 μm). Signals were amplified 10,000× and filtered between 500 Hz and 3.5 kHz for extracting multi-unit (MU) activity. The waveforms of detected spikes were recorded for a duration of 1.2 ms, which allowed the later application of offline spike-sorting techniques to extract single units (SU).

### Datasets

One dataset (col05-e08) was recorded in response to sinusoidal gratings moving in 12 directions in steps of 30°, presented in a randomized order in trials of 4800 ms (1000 ms spontaneous activity, 3500 ms stimulus, 300 ms OFF-response). Gratings had a spatial frequency of 2.4° visual angle per grating cycle, moved at a speed of 2° per second and spanned 12° in the visual field. Analyses were conducted on 26 simultaneously recorded SUs with overlapping receptive fields. Two other datasets (col11-b44 and col11-b68) were recorded in response to sinusoidal gratings of three different sizes. Gratings were presented either one-by-one or superimposed (a smaller grating displayed on top of and in the center of a larger one) (Biederlack et al., 2006). When overlapping, gratings had the same or orthogonal orientations and in some cases they were segregated by a gray ring. Gratings extended over visual angles of 7, 14, and 21°, had a spatial frequency of 1° per grating cycle and were presented at a speed of 1.5° per second. The resulting 14 stimuli were presented 20 times each in a randomized order in trials of 6000 ms long (stimuli shown between 1000 and 5000 ms). Analyses were performed on 12 SUs that were identified in both datasets.

### Data Analysis

Simultaneously recorded spike trains can be represented as vectors in a high-dimensional space, where each dimension corresponds to a neuron. A vector element describes the spiking activity of a neuron in a given time window, while an entire vector describes the spiking of all recorded neurons during that time window. Quantifying a neuron’s activity within a time window can be achieved trough binning its spikes (Grün et al., 2002; Schneidman et al., 2006; Osborne et al., 2008). Another option, pioneered by Gerstein and Aertsen (1985), is to convolve spikes with exponentially decaying kernels (Nikolić et al., 2007; Häusler and Maass, 2007; Jurjut et al., 2009, 2011) and sampling the resulting signal. We chose the latter approach since exponentially decaying kernels have a biological correspondent, namely the post-synaptic currents (Jurjut et al., 2009, 2011).

#### Convolving Spike-Trains

**FIGURE 1:**
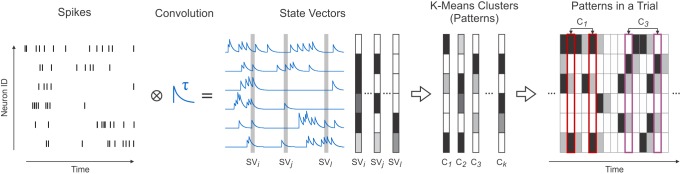
Transforming spikes into state vectors and their subsequent clustering. Simultaneously recorded spike trains (Spikes) convolved with exponentially decaying kernels (Convolution) were sampled to extract activity vectors (State Vectors). Using a K-Means algorithm, state vectors were clustered (K-Means Clusters) and replaced in the data with their corresponding clusters (patterns) (Patterns in a Trial).

Spike trains were convolved using an exponentially decaying kernel (Figure 1, Convolution). The activity of a neuron *i*, was transformed into a continuous signal *s_i_*(*t*) using the formula:

$$s_{i}(t)=\left\{ \begin{array}{c} s_{i}(t-1)+1,\text{if neuron}\,\;i\,\;\text{has a spike at time}\;t\\ s_{i}(t-1)\cdot e^{-\frac{1}{\tau }},otherwise \end{array}\right.$$

where, *s_i_*(*t*) is the signal of neuron *i* after convolution and *τ* is the convolution kernel’s decay (integration) time constant. To be consistent with the known range of values for neuronal membrane time constants, in our analyses we used *τ* = 20 ms (Spruston and Johnston, 1992; Kasper et al., 1994; Magee, 1998), except when stated otherwise. After convolution, the multiple signals resulting from the simultaneously recorded spike trains were sampled with a frequency of 1 kHz and for each time bin a vector was constructed:

$$SV(t)=\left[s_{1}(t),s_{2}(t),...,s_{n}\left(t\right)\right]$$

where, *n* is the number of analyzed neurons. *SV*(*t*) can be interpreted as a “snapshot” of the state of all convolved spike trains at time *t* (Figure 1, State Vectors). Hence, *SV* was termed *state vector*.

#### Clustering

Techniques that involve binarization and bining of spikes are convenient because they allow the identification of a limited number of firing patterns (2*^n^*; Schneidman et al., 2006; Osborne et al., 2008). In our case, however, because we have used exponentially decaying kernels instead of binarization, each entry of a state vector takes values in a continuous interval. Therefore, one cannot define individual patterns as is the case for the binary representation, since there is an infinite number of possible state vectors. To identify classes of state vectors that occur robustly throughout a recording session, we used a simple K-Means clustering algorithm (Lloyd, 1982) with *K* = 1000 and Euclidean metric. Initially, state vectors (samples) were randomly assigned to clusters, which were computed afterward based on their corresponding samples:

$$C_{i}[j]=\frac{\Sigma_{l}^{L_{i}}{SV}_{l}\left[j\right]}{L_{i}};i=\overset{¯}{1,K}\text{ j=}\overset{¯}{1,n}$$

where *K* is the number of clusters, *n* is the neuron count, *L_i_* is the number of samples assigned to cluster *C_i_*, and *SV*_l_ denotes a sample assigned to cluster *C*_i_. Each iteration consisted of reassigning samples to their nearest cluster (the one with smallest Euclidian distance to the sample) and recomputing all clusters. At each step *s* the approximation error *E_s_* was computed as:

$$E_{s}=\overset{K}{\underset{i}{\Sigma }}\overset{L_{i,s}}{\underset{l}{\Sigma }}\sqrt{\overset{n}{\underset{j}{\Sigma }}{\left(C_{i,s}[j]-{SV}_{l}\left[j\right]\right)}^{2}}$$

where *C*_i,s_ denotes cluster *i* at step *s*, *L*_i,s_ is the number of samples assigned to *C*_i,s_ and *SV*_l_ denotes a sample assigned to cluster *C*_i,s_. Iterations were repeated until the difference between *E*_*s*-1_ and *E*_*s*_ was smaller than ε^⋅^*E*_*s*-1_ (ε = 0.01 in our analysis). The resulting *K* clusters approximated the spiking patterns of all recorded neurons (Figure 1, K-Means Clusters). Therefore, clusters will further be referred to as *patterns*. We chose this algorithm for simplicity purposes. Other clustering algorithms may yield better performance in approximating this type of data. However, the simple K-Means algorithm used here provided a sufficiently accurate representation of the data and enabled us to identify robustly occuring patterns.

After clustering, state vectors were replaced with their corresponding patterns (clusters). Hence, a recorded trial was described as a sequence of individual patterns appearing in successive bins of 1 ms (Figure 1, Patterns in a Trial). All subsequent analyses were performed using this representation of the data.

#### Active and Silent Neurons

In a pattern, an entry corresponding to a neuron can have a large value, indicating that the neuron spiked recently, or a small value, indicating a lack of spikes in the recent past. Using a threshold of 0.36 (∼1/*e*), we defined each neuron as being *active* (≥0.36) or *silent* (<0.36) in a pattern. This threshold was chosen as follows: For a single isolated spike at time *t*, the convolved spike-train’s value (Figure 1) decays from a value of 1.0 to ∼0.36 after *τ* ms (see Eq. 1). Other thresholds may be chosen, but our experience has shown that thresholds <0.36 do not produce any notable differences in the results.

#### Smoothed Peri-Stimulus Time Histograms

The PSTH for a neuron/pattern was smoothed using a rectangular sliding window of size 2*h*+1, and, given a stimulus *j*, was defined as follows:

$${PSTH}_{j}(t)=\frac{\Sigma_{l}\frac{r_{l}\left(t-h,t+h\right)}{2h+1}}{T_{j}};l=\overset{¯}{1,T_{j}}$$

where, *t* represents the time in the trial, *h* is half the size of the rectangular smoothing window, *r*_l_(*t-h*, *t+h*) is the spike/pattern count in window [*t-h*, *t*+*h*] of trial *l*, and *T*_j_ is the number of trials recorded with stimulus *j*. In our analyses we used *h* = 100 ms for both neurons and patterns.

#### Direction and Orientation Tuning

Tuning curves were computed as histograms of spike/pattern occurrences per stimulus. To quantify direction and orientation tuning to gratings, we applied the measures of Direction Index (DI) and Orientation Index (OI) (Kida et al., 2005):

$$DI=\sqrt{{\left[\Sigma \left(r_{j}\cdot \sin\theta_{j}\right)\right]}^{2}+{\left[\Sigma \left(r_{j}\cdot \cos\theta_{j}\right)\right]}^{2}}\,/\Sigma r_{j}$$

$$OI=\sqrt{{\left[\Sigma \left(r_{j}\cdot \sin{2\theta }_{j}\right)\right]}^{2}+{\left[\Sigma \left(r_{j}\cdot \cos{2\theta }_{j}\right)\right]}^{2}}\,/\Sigma r_{j}$$

where, *r*_j_ is the spike/pattern count in response to stimulus *j* and θ_j_ is the angle for the direction of movement of the respective stimulus. DI and OI take values between 0 and 1; 0 when the spikes/patterns occur equally in all stimuli, and 1 when they appear only for one stimulus (DI) or only for two diametrically opposed stimuli (OI).

#### Auto and Cross-Correlograms

Auto and cross-correlograms were computed as histograms of spike/pattern coincidences at various time shifts. Suppose *f*(*t*) and *g*(*t*) are two equally long binary signals that can take values of 0 (no spike/pattern occurs at time *t*) or 1 (a spike/pattern occurs at time *t*). Their cross-correlation histogram (*CCH*) at a time lag δ is:

$${CCH}_{f,g}(\delta )=\overset{T}{\underset{t}{\Sigma }}f(t)\cdot g\left(t+\delta \right)$$

where *T* is the length of signals and δ takes values from [*-h*,*h*], where *h* is the size of the correlation window. The auto-correlation histogram (*ACH*) of a signal *f*(*t*) is simply the *CCH* of the signal with itself:

$${ACH}_{f}(\delta )=\overset{T}{\underset{t}{\Sigma }}f(t)\cdot f\left(t+\delta \right)$$

In our analyses we used *h* = 100 ms when computing *ACH*s and *CCH*s for both neurons and patterns, thus focusing on small lags, i.e., fast processes (Nikolić et al., 2012).

## Results

The occurrence of a pattern is a binary event, similar to a spike, therefore measures applied to spike trains, such as tuning curves, peri-stimulus time histograms, auto- and cross-correlograms, are directly applicable to patterns. We used these measures to investigate pattern behavior and compared it to the behavior of single neurons active within these patterns (see sections “Methods” and “Data Analysis”). Examples were selected from three datasets: one consisting of responses of 26 single units evoked with drifting sinusoidal gratings and two consisting of responses of 12 single units evoked with center-surround gratings (see sections “Methods” and “Datasets”). In all subsequent figures, patterns are represented as vertical bars that use the grayscale to code the activation of each neuron (white = silent, black = active).

**FIGURE 2:**
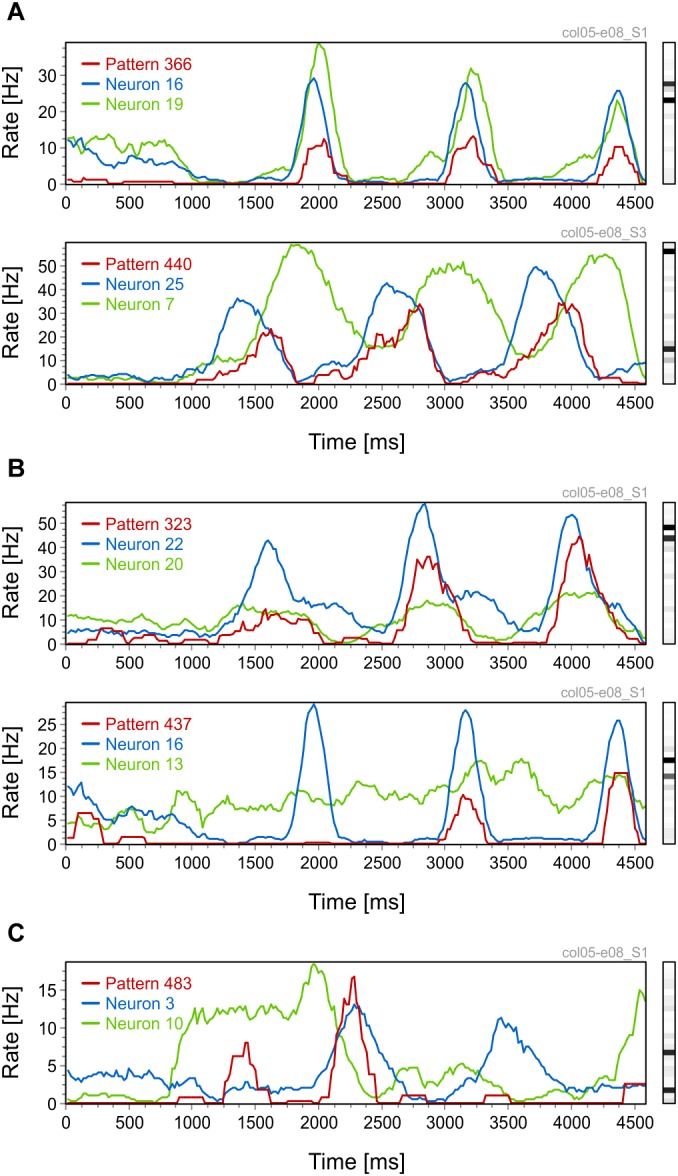
Peri-stimulus time histograms (PSTHs) for patterns and their active neurons. **(A)** Pattern PSTHs follow the PSTHs of their active neurons. **(B)** Patterns with increasingly higher PSTH peaks toward the end of the trial. **(C)** A pattern PSTH that has no apparent relation to the PSTHs of its active neurons. Each figure panel is labeled on top (gray text) with the code of the dataset and the corresponding stimulation condition.

We first investigated the stimulus-locking of pattern occurrences and compared it to the stimulus-locking of their active neurons. Thus, for both patterns and neurons we computed peri-stimulus time histograms (PSTH), smoothed with a 200 ms time window, in response to sinusoidal grating stimuli. Intuitively, one expects a high rate of pattern occurrence whenever the constituting active neurons have high spike rates. Figure 2A shows such examples: one where the PSTH of the pattern follows the PSTHs of the neurons (top row) and one where the shape of the pattern PSTH faithfully reflects the coactivation of the two constituting active neurons. The three peaks in the PSTHs correspond to the periodic activity modulation induced by the drift of the sinusoidal grating. Patterns can also exhibit a behavior that cannot be intuitively predicted from the activity of the constituting active neurons. In Figure 2B one can observe similar modulations in the pattern PSTH as in Figure 2A, but the amplitude of these modulations changes throughout the trial. Modulations that are initially small (Figure 2B, top row) or absent (Figure 2B, bottom row) become amplified toward the end of the trial. Notice also that the amplitude of the PSTH peaks varies less for neurons than for patterns. Another interesting example is shown in Figure 2C where a pattern with two active neurons exhibits a peak in the PSTH at around 1450 ms that cannot be predicted from the PSTHs of active neurons.

**FIGURE 3:**
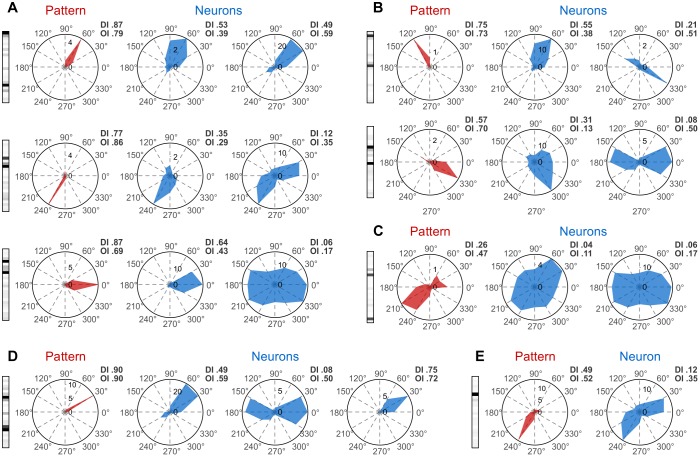
Tuning properties of patterns. Pattern tuning curves (TCs) are shown on the left side of each panel in red, while TCs of their active neurons are shown in the same row in blue. **(A)** Patterns with similar direction preference as their active neurons. **(B)** Patterns with a direction preference intermediate to those of their active neurons. **(C)** A sharply tuned pattern composed of two active neurons that have poor tuning. **(D)** A tuned pattern with three active neurons. **(E)** A pattern having a TC different from that of its only active neuron. Direction Tuning Index (DI) and Orientation Tuning Index (OI) are displayed in the upper-right corner of each TC.

Next, we examined whether patterns can be tuned to the direction and orientation of stimuli, as has been shown for individual neurons in the primary visual cortex (Hubel and Wiesel, 1962). For both neurons and patterns we computed tuning curves (TCs) and indexes of orientation (OI) and direction (DI) tuning, which characterize the degree of tuning of a neuron/pattern to orientation or direction, respectively (Kida et al., 2005) (see sections “Methods” and “Direction and Orientation Tuning”). Figure 3A, depicts TCs corresponding to three example patterns (Figure 3A, red), each having two active neurons. In this example, active neurons (Figure 3A, blue) had similar but broad tuning preferences. Patterns had the same orientation/direction preference as the neurons, but the tuning of the former was usually much sharper. Figure 3B shows two examples of patterns, each with two active neurons having slightly different orientation preferences. The corresponding patterns exhibit again a sharper tuning, but with preference for an intermediate orientation (120° top row, 330° bottom row). The increase in sharpness of tuning is not limited to patterns generated by well tuned active neurons. Figure 3C shows an example in which two neurons with poor tuning produce a more sharply tuned pattern. Sharpness of tuning can also be higher than that of individual neurons for patterns consisting of more than two active neurons. Figure 3D shows such an example where three neurons with broad but similar tuning preferences produce a very sharply tuned pattern. Finally, tuning properties of patterns cannot always be predicted from the tuning of the active neurons. Figure 3E shows such an example of a pattern that has only one active neuron while the rest are silent. The TC of the active neuron reveals a preference for 30° and 240° while the TC of the pattern shows only a preference for 240°, which is in addition more sharply tuned than the neuron’s spiking preference for 240°. Thus, silent neurons can contribute also to the sharpening of the tuning of patterns (see section “Discussion”).

**FIGURE 4:**
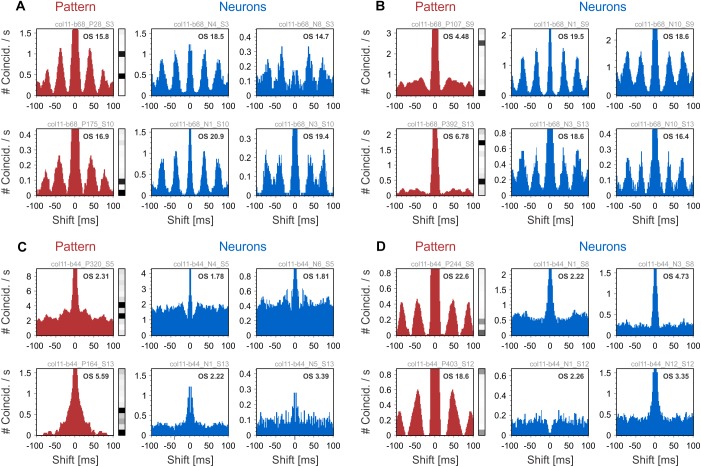
Auto-correlation histograms (ACHs) of patterns and their active neurons. ACHs of patterns are shown in red on the left side of each panel, while ACHs of their active neurons are shown in blue on the right. **(A)** ACHs of patters showing oscillatory activity in the same frequency range as the pattern’s active neurons. **(B)** Patterns that show no oscillatory activity, although the ACHs of their active neurons exhibit oscillations. **(C)** Non-oscillating patterns that have non-oscillating active neurons. **(D)** ACHs of patterns showing oscillatory behavior even though their active neurons are not oscillating. The Oscillation Score (OS) of each ACH is displayed in the upper-right corner.

We next show examples of the oscillatory behavior of patterns and their comparison to the oscillatory behavior of their active neurons. To this end, we used auto-correlation histograms (ACH) and quantified oscillatory behavior by computing oscillation scores (OS) in the high-beta/low-gamma bands (20–50 Hz; Mureşan et al., 2008). We used two datasets containing responses to center-surround gratings (see sections “Methods” and “Datasets”). In one dataset, neurons exhibited robust gamma-band oscillations (average OS 9.84), while in the other dataset oscillations were poor or absent (average OS 3.63). Examples in Figure 4A,B were chosen from the dataset with oscillatory responses (col11-b68), while the ones in Figure 4C,D were selected from the dataset with non-oscillatory responses (col11-b44). Figure 4A shows two examples of patterns with oscillatory modulation in the same frequency band as the oscillatory responses of the active neurons. In Figure 4B the patterns do not exhibit oscillatory activity although their active neurons have strong oscillatory responses. Such behavior may arise if the oscillations in the component neurons are not phase-locked and therefore their co-firing occurs in a non-periodic manner. In Figure 4C the ACHs of patterns and their active neurons reveal no oscillations. In contrast, Figure 4D shows patterns whose expression is markedly oscillatory even though the responses of the active neurons show no oscillatory modulation. Oscillatory activity of silent neurons could possibly explain this phenomenon (see section “Discussion”).

As a control, we tested whether the oscillatory behavior of patterns was an artifact of the convolution of spikes with exponentially decaying kernels (time constant of 20 ms). Oscillations were present in the pattern ACHs also at smaller integration time constants (1, 5, and 10 ms; results not shown), indicating that the observed oscillatory structure is not an artifact of convolution. Overall, examples in Figure 4 suggest that inferring the oscillatory behavior of patterns from the activity of their active neurons is not straightforward.

**FIGURE 5:**
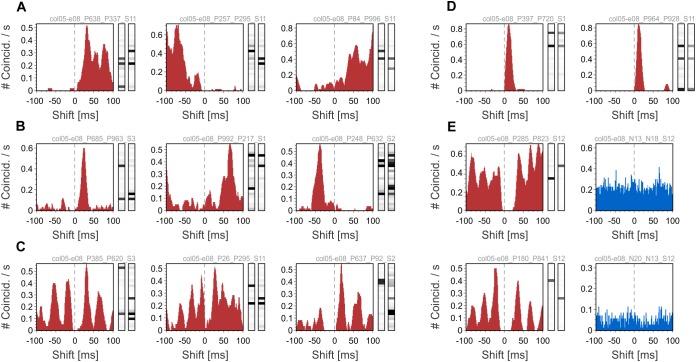
Cross-correlation histograms (CCHs) of patterns. **(A)** Patterns that follow each other over a broad time interval. **(B)** CCHs of pattern that occur predominantly at a specific time interval. **(C)** CCHs of patterns that follow each other at regular time intervals. **(D)** Patterns with the same active neurons, but at different activation levels, occurring one after the other at short time lags. **(E)** Two examples of CCHs between patterns each having one active neuron (left, red) and CCHs between these active neurons (right, blue).

Using cross-correlation histograms (CCH), we illustrate on the dataset with simple gratings (col05-e08) how one can investigate whether there are preferences in the relative occurrences of different patterns. Note that, by definition, two different patterns cannot occur at the same time (i.e., zero-lag coincidence is not possible). Figure 5A shows three examples in which distinct patterns follow each other over a broad range of time-lags. Examples in Figure 5B show patterns that occur in a sequence at a preferred time interval (∼24 ms, left panel; ∼68 ms, center panel; ∼-40 ms, right panel). In Figure 5C pattern CCHs exhibit multiple peaks, indicating multiple preferred lags between the patterns. In Figure 5D patterns with the same active neurons that are more activated in one pattern than in the other, follow each other at short time lags (∼12 ms). The “faded” pattern (the one with less activated neurons) follows the other as a consequence of convolution with exponentially decaying kernels (see sections “Methods” and “Data analysis”). The two patterns represent the same set of spikes, but at slightly different times. This effect must be taken into consideration when interpreting the results. Finally, in Figure 5E we show two examples of CCHs between patterns (left panels), each having one active neuron, and the CCHs between their corresponding active neurons (right panels). While pattern CCHs exhibit peaks at regular time intervals, CCHs of the neurons’ spiking activity do not exhibit any related time structure (blue). This could result from the fact that patterns are more constrained than their active neurons, i.e., a pattern with one active neuron can only appear when all the other neurons are silent.

Finally, we uses local-field potential (LFP) signals to probe deeper into circuit mechanisms that underlie coordinated firing or silence of neurons. We selected units from dataset col05-e08 that matched the following criteria: (i) they could be reliably identified as single units; (ii) they had overlapping tuning curves for at least one grating direction (i.e., similar tuning); (iii) each unit was isolated from a different recording electrode. Seven units exhibited this property, firing preferentially in response to gratings drifting at 330°. The rationale for this selection was to observe the timing coordination between units that fire in response to the same stimulus and to determine their relation to the corresponding meso-scale dynamics, i.e., LFPs. The latter may provide information about the subthreshold state of silent units under conditions of high spike-field coherence arising from correlated population dynamics (Denker et al., 2011). To avoid temporal smearing and to match LFP timescales, we computed patterns on the activity of the seven units using a fast time constant, *τ* = 5 ms. All other parameters were unchanged (see section “Methods”).

**FIGURE 6:**
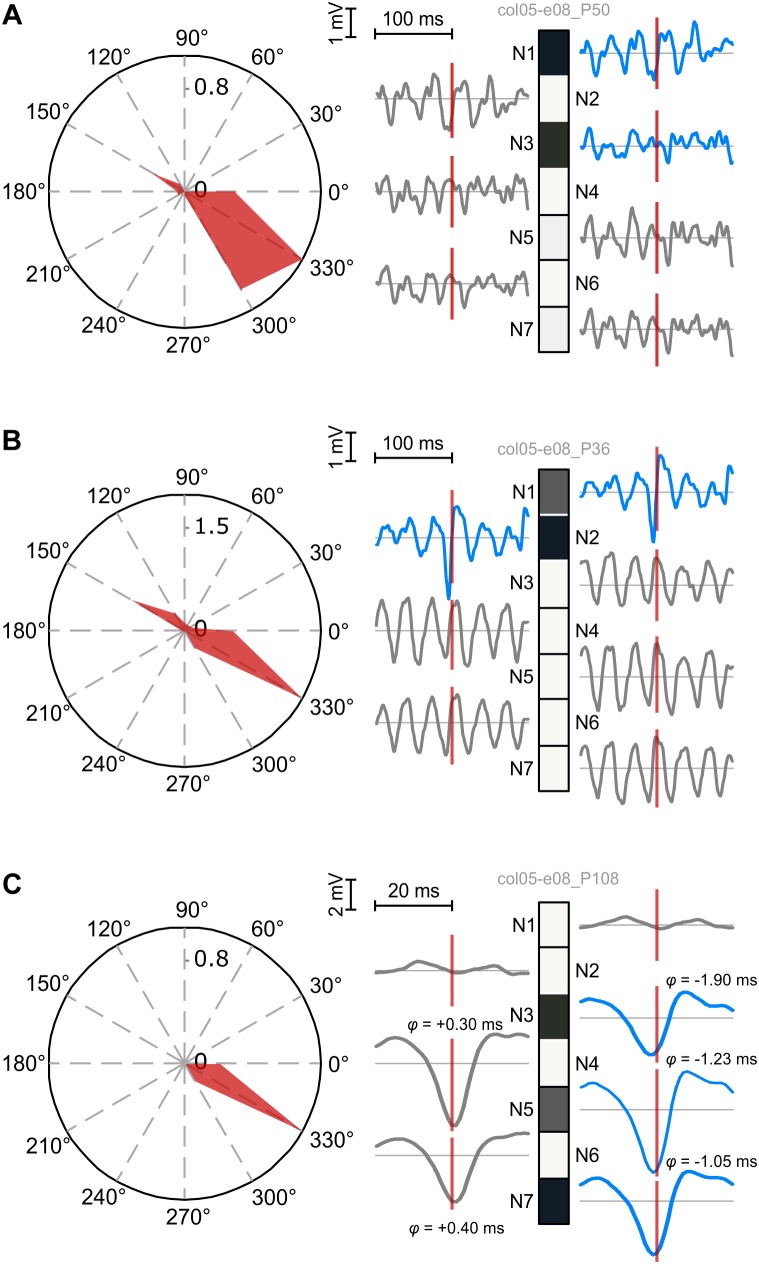
Temporal coordination of active and silent units contributing to patterns. Left: pattern tuning curves, showing preferential selectivity to 330° drifting gratings. Right: patterns and the corresponding pattern-triggered LFP average (PTA) computed for each electrode of source units. Blue traces correspond to PTAs computed on LFPs from electrodes with active units. **(A)** Fuzzy coordination between pattern expression and LFPs. No consistent rule can be derived for PTAs using LFPs from active and inactive electrodes. **(B)** Coherent firing and silence of two subpopulations (N1 and N2 vs. N3–N7) is reflected in precise alignment of the pattern to excitatory troughs and inhibitory peaks of corresponding LFPs. Note the prominent oscillatory modulation of LFPs from silent electrodes. **(C)** Fine, sub-millisecond temporal coordination of firing producing a pattern with three active neurons. LFPs corresponding to active neurons tend to trough before pattern expression, while those corresponding to silent neurons trough after it. The temporal delay was estimated by computing the offset at half minimum of the trough.

Patterns generated by the seven neurons exhibitted a variety of behaviors relative to the LFPs recorded on the source electrodes. As expected, patterns were also selective to 330° drifting gratings (Figure 6). We correlated the expression of patterns with the LFP corresponding to each component neuron’s source electrode by computing the pattern-triggered LFP average (similar to the spike-triggered average; see Gray and Singer, 1989; Nikolić et al., 2012). We next show three typical examples. Figure 6A depicts a pattern (P50) whose alignment to LFPs associated to active and inactive neurons (units) was not systematic: P50 was aligned to the LFP trough (N1 and N2), LFP peak (N4–N7), or not clearly aligned (N3).

Figure 6B illustrates a pattern (P36) whose alignment to the LFP was much sharper and coherent. P36’s expression was sharply locked to the trough of the active units’ corresponding LFP, reflecting the spike volley that generated the pattern. This sharp negative LFP deflection suggests a correlated excitatory state at the level of the local circuits in which active neurons were embedded (Denker et al., 2011). On the other hand, P36 was aligned to the peaks of LFPs corresponding to silent neurons. The LFP peak likely reflects a period with lingering inhibition generated during the previous trough-aligned volley (Teleǹczuk et al., 2017). Indeed, LFPs on silent electrodes displayed marked oscillatory entrainment in the gamma band, therefore peaks of the LFP corresponded to the inhibited (down-state) phase in the oscillation. P36 thus reflects a complex coordination of two subnetworks: N1 and N2 fired in coordination while, at the same moments in time, N3–N7 were coherently silenced by an offset gamma rhythm.

Finally, in Figure 6C we identified a pattern (P108) with three active neurons, sharp selectivity, and strong correlation to the LFPs (note the amplitude scale). We zoomed in on the central part of the pattern-triggered average to identify the fine temporal details (note the temporal scale). Consistently with the other two examples, LFPs corresponding to N1 and N2 had a different behavior than those corresponding to the other neurons: in this case their LFPs were not correlated to P108’s expression. On the other hand, LFPs corresponding to the remaining five neurons exhibitted an interesting property: those associated to active neurons tended to trough before P108’s expression (negative lag), while those associated to silent neurons troughed after P108’s manifestation. These results indicate very fine coordinated fluctuations in the sub-millisecond range, whereby active neurons were engaged right before pattern expression, thus contributing to it, while the silent neurons were slightly delayed, providing a narrow window of opportunity for the manifestation of the pattern.

## Discussion

Many studies have investigated neuronal population dynamics (Friedrich and Laurent, 2001; Galán et al., 2004; Brown et al., 2005; Bathellier et al., 2008) and it has been shown that multi-neuron activity carries more stimulus related information than individual cells (Johnson, 2004; Biederlack et al., 2006; Schneidman et al., 2006; Osborne et al., 2008). Here we describe the collective behavior of simultaneously recorded neurons as multidimensional vectors, called patterns, and subject the latter to classical spike train analyses. We have shown that pattern behavior can exhibit a wide range of properties, either similar to those of individual constituting neurons, or considerably different. Moreover, pattern behavior cannot always be inferred from that of its active neurons. Importantly, however, silent neurons define a pattern too, and hence, the expression of the pattern depends on both the activation and lack of activation of its respective neurons. Indeed, it was shown that silent neurons also contribute to coding (Osborne et al., 2008) and that non-classically responding units participate in ensembles carrying stimulus-related information (Insanally et al., 2019). For example, a pattern composed of active neurons that do not show oscillatory responses may exhibit oscillatory behavior simply because one neuron, contributing as “silent” to the pattern, fires with an oscillatory rhythm. This neuron is silenced at regular intervals, constraining the occurrences of the pattern to those intervals and thus it imposes an oscillatory modulation on the occurrence of the pattern. The same principle applies to PSTHs. Activity of neurons that are contributing as silent to a pattern creates intervals in which the pattern cannot be expressed. The outcome is that the pattern’s PSTH can be considerably different from the PSTHs of the contributing active neurons. Therefore, both active and silent neurons have to be taken into account when investigating the expression of a neuronal pattern. The definition of multidimensional activity patterns includes different effects: Active neurons are a sign of excitation while the silence of neurons in a pattern could result from lack of excitation or even from inhibition. All these effects will contribute to the expression of the respective pattern, and hence, the rich interaction of these multiple mechanisms can be harvested in full with the present approach.

We have shown that the role of silent neurons in constraining the expression of patterns is just as important as the role of actively firing units. Silent neurons can be coordinated precisely by subthreshold dynamics dictated by the activity of the embedding mesoscale, local circuit, whose state can be inferred from the corresponding LFP. We found several typical relations between pattern expression and the LFPs corresponding to its active and silent units. Sometimes a systematic relationship is lacking, but in many other cases patterns arise from coordinated activation and silencing of neurons, involving oscillatory modulation and precise temporal offsets with sub-millisecond precision (Havenith et al., 2011). Results indicate that correlating pattern expression with LFPs allows one to also disect multiple cortical subnetworks, whose dynamics are precisely orchestrated within and across, giving rise to a rich repertoire of patterns.

In visual cortex oscillations can orchestrate circuit dynamics in both the beta and gamma bands (Bastos et al., 2015). Here, we have observed oscillations around 27 Hz, i.e., in the beta-high band (20–30 Hz), at the border with gamma (30–80 Hz). As discussed elsewhere, the beta-high and gamma bands are sometimes indistinguishable (Mureşan et al., 2008) and according to some studies they are termed together, simply as gamma (Whittington et al., 1997). Lower gamma frequencies observed in the present preparation, spilling into the beta-high band, fluctuate during anesthesia, visiting both gamma and beta bands, while also depending on the properties of the visual stimuli (Feng et al., 2010). Irrespective of their frequency, oscillations >20 Hz, whose period approaches the membrane time constants, most likely contribute to the dynamical organization of firing and quiescence that defines specific neural ensembles and their properties. From a technical point of view, we have introduced an approach that uses classical, established methods, which are well understood analytically. These methods provide experimentalists with a familiar working environment and can be equally well applied to both patterns and the spiking of individual neurons, making it easy to evaluate the degree of similarity between the behavior of individual neurons and that of their corresponding activity patterns. This can also help us understand how patterns emerge from the activity of individual neurons.

Our goal was to present a methodology without formulating any conclusions on putative neuronal codes or their readout. We have provided a few examples illustrating the extent to which pattern expression can differ from the spiking responses of the constituting neurons. When investigating putative strategies of distributed coding, classical analysis techniques as described in this study, might become a valuable tool. They produce comprehensive quantitative descriptions of population activity that are amenable to rigorous statistical testing.

## Data Availability

The datasets for this manuscript are not publicly available because Datasets were recorded in the Max Planck Institute for Brain Research before 2008. Our manuscript does not use this data to draw any conclusion about brain function but merely to exemplify the novel methodology developed. The particular datasets used are irrelevant for the message of the manuscript and not needed in any way for the reproduction of the methodological steps described in the manuscript. Requests to access the datasets should be directed to danko.nikolic@googlemail.com.

## Ethics Statement

All experiments were conducted in accordance with the Society for Neuroscience, German law, and the European Communities Council Directive of 24 November 1986 (86/609/EEC) regarding the care and use of animals for experimental procedures, approved by the local government’s ethical committee (Regierungspräsidium Darmstadt), and overseen by a veterinarian.

## Author Contributions

OJ, MG, and RM analyzed the data. DN recorded the data. WS provided the research infrastructure and contributed to writing the manuscript. RM coordinated the method development and data analysis and wrote the manuscript.

## Conflict of Interest Statement

DN was employed by company Savedroid AG, Frankfurt am Main, Germany. The remaining authors declare that the research was conducted in the absence of any commercial or financial relationships that could be construed as a potential conflict of interest.
